# Supplementary material for: Nearest neighbor search on embeddings rapidly identifies distant protein relations
Source: Front Bioinform. 2022 Nov 17;2:1033775. doi: 10.3389/fbinf.2022.1033775 (PMC9714024; doi:10.3389/fbinf.2022.1033775)
Supplement: Supplementary file 1 [file DataSheet2.PDF]

Supporting online material for: Nearest neighbor search on embeddings rapidly identifies distant protein relations

Konstantin Schütze[1,*], Michael Heinzinger[1,2], Martin Steinegger[3], Burkhard Rost[1,4]

1 TUM (Technical University of Munich) Department of Informatics, Bioinformatics & Computational Biology - i12, Boltzmannstr. 3, 85748 Garching/Munich, Germany
2 TUM Graduate School, Center of Doctoral Studies in Informatics and its Applications (CeDoSIA), Boltzmannstr. 11, 85748 Garching, Germany
3 School of Biological Sciences, Seoul National University, Seoul, South Korea & Artificial Intelligence Inst, Seoul National University, Seoul, South Korea
4 Institute for Advanced Study (TUM-IAS), Lichtenbergstr. 2a, 85748 Garching/Munich, Germany & TUM School of Life Sciences Weihenstephan (WZW), Alte Akademie 8, Freising, Germany
* Corresponding author: Konstantin Schütze, schuetze@in.tum.de ; email Rost <assistant@rostlab.org>

Table of Contents for Supporting Online Material

SUPPORTING ONLINE MATERIAL FOR: NEAREST NEIGHBOR SEARCH ON EMBEDDINGS RAPIDLY IDENTIFIES DISTANT PROTEIN RELATIONS 1

TABLE OF CONTENTS FOR SUPPORTING ONLINE MATERIAL 1

SHORT DESCRIPTION OF SUPPORTING ONLINE MATERIAL 2

T5 Detecting Shuffled Sequences 2

MATERIAL 3

Fig. S1: CATH20 raw accuracy by E-value cutoff 3
Fig. S2: CATH20 normalized accuracy by E-value cutoff 4
Fig. S3: SeqVec layers for CATH20 5
Fig. S4: CATH20 E-Value vs. Cosine Similarity 6
Fig. S5: Pfam20 accuracy by cosine similarity cutoff 7
Fig. S6: Influence of number of hits for Pfam20 8
Fig. S7: Comparing shuffled and reversed sequences 9
Table S1: Euclidean vs. cosine CATH20 accuracies 11
Table S2: Separating fake sequences by PC1 12

REFERENCES FOR SUPPORTING ONLINE MATERIAL 13

Short description of Supporting Online Material

In the main, we show the combined MMseqs2 + ProtT5 k-nn method on CATH20 only for an example cutoff of E<0.1 (Steinegger and Söding, 2017, Elnaggar et al., 2021, Orengo et al., 1997). Here we add figures for the whole range of E-value (Fig. S1 and Fig. S2 for *QrawTop1* and *QnormTop1*). Fig. S3 show how unlike the more complex linear combinations in ELMo (Peters et al., 2018), SeqVec for us performs best best when just using the hidden state from the LSTM1 layer. Table S1 shows how the cosine similarity beats the euclidean distance consistently. In Fig. S4, we confirm the low correlation between E-Value and cosine similarity.

For the Pfam20 dataset (El-Gebali et al., 2019), we show the correlation between cosine similarity and accuracy (Fig. S5) and how the number of hits we allow in a prefilter influences the fraction of homologs retrieved (Fig. S6). We can't shuffle unannotated regions in proteins as other benchmarks did (Steinegger and Söding, 2017, Buchfink et al., 2021) since ProtT5 can trivially separate real and fake sequences (Following section, Fig. S6 and Table S2)

T5 Detecting Shuffled Sequences

Real sequences vs. shuffled sequences ProtT5 can tell real sequences from reversed and shuffled sequences, generating considerably different embeddings. This can be shown by even only looking at principal component 1 of a PCA of the embeddings of native proteins and scrambled counterparts. As an example, we took 10000 random proteins from the Pfam set, generated embeddings for each original sequence, each sequence randomly shuffled and each sequence reversed. We project original and shuffled embeddings as well as original and reversed embeddings in a PCA. Supplementary Fig. S7 shows the distribution on embeddings across Principal Component 1. Using a cutoff of 0 with the values from this axis, this can be seen as a basic predictor for real vs. scrambled, for which we show confusion matrices in Supplementary Table 2. Surprisingly, we even get a good split without perform any kind of transformation by only taking embedding dimension 519, marking everything below 0.15 as real and everything above as scrambled. This alone reaches an accuracy of over 85% for each class on the tested set. This shows that ProtT5 can differentiate between real and "fake" sequences and that "fakeness" is the primary signal. This is also not due ProtT5 misses the methionine at the beginning of a protein as the same behaviour occurs with the domains from the CATH dataset.

Material

Fig. S1: CATH20 raw accuracy by E-value cutoff

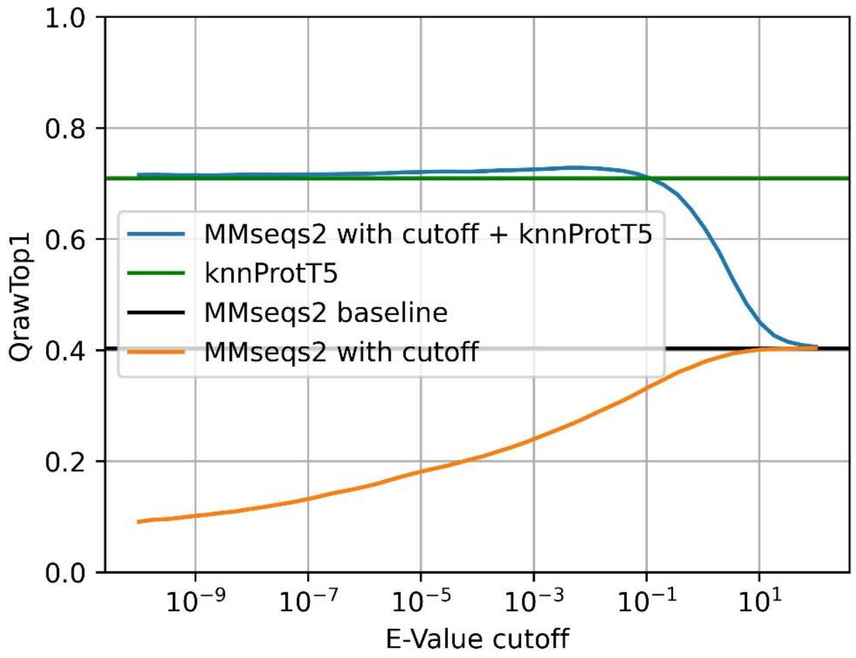

Fig. S1: CATH20 raw accuracy by E-value cutoff. The QrawTop1 accuracy of MMseqs2 {Elnaggar, 2021 #66} and the combined approach at different E-value cutoffs when searching for domains from the same homologous superfamily in CATH {Orengo, 1997 #74;Sillitoe, 2019 #49}. The blue line is the novel combined method, the green line is the proposed nearest neighbor search with ProtT5 XL U50 {Elnaggar, 2021 #66} and the black line is the MMSeqs2 baseline. The orange line is what we would get if we took the combined method and removed all the k-nn hits, which shows which fraction of the combined method score comes from MMseqs2 and which from k-nn. Equivalent of Fig. 2 with raw accuracies.

Fig. S2: CATH20 normalized accuracy by E-value cutoff

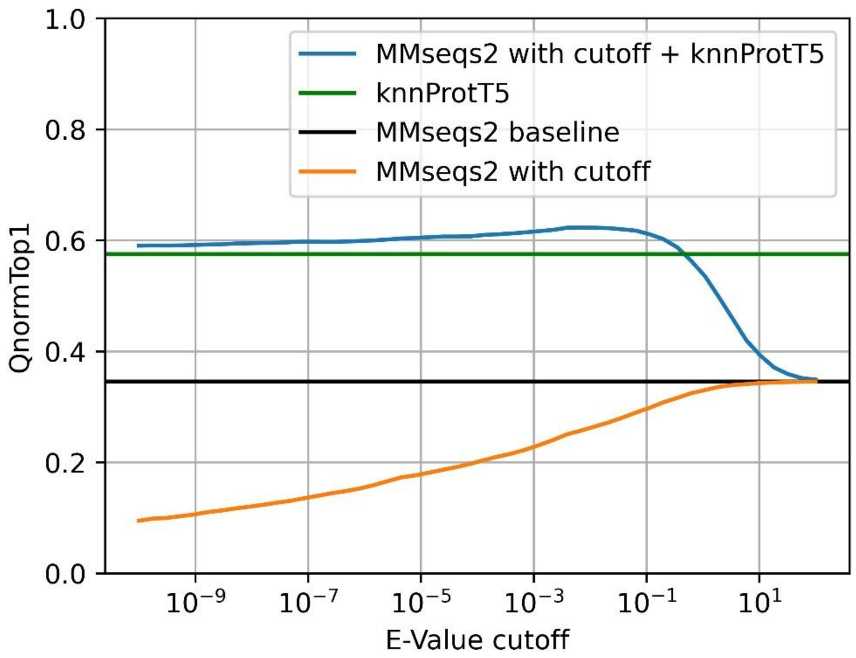

Fig. S2: CATH20 normalized accuracy by E-value cutoff. Identical to Fig. S1, except with QnormTop1 instead of QrawTop1

Fig. S3: SeqVec layers for CATH20

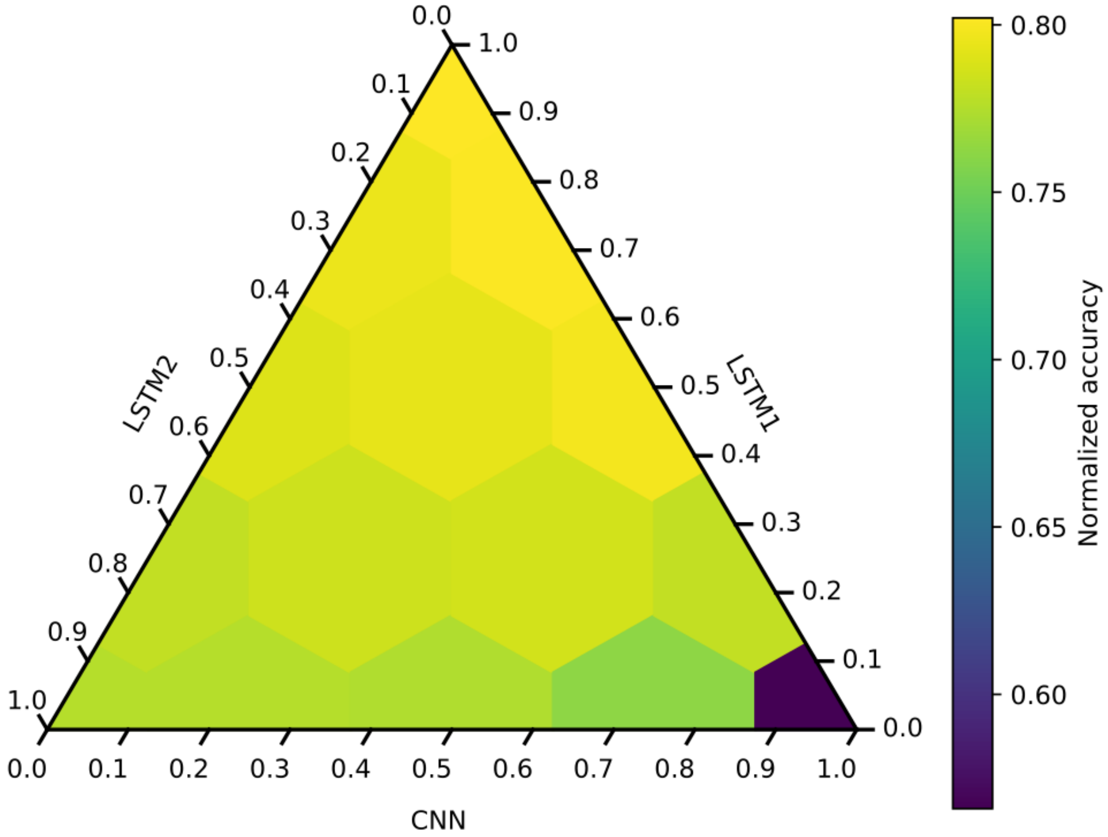

Fig. S3: SeqVec layers for CATH20. QnormTop1 on the CATH dataset for different linear combinations of SeqVec layers as ternary plot, showing how LSTM1 alone is the best layer choice, while combinations of layers do not improve over it.

Fig. S4: CATH20 E-Value vs. Cosine Similarity

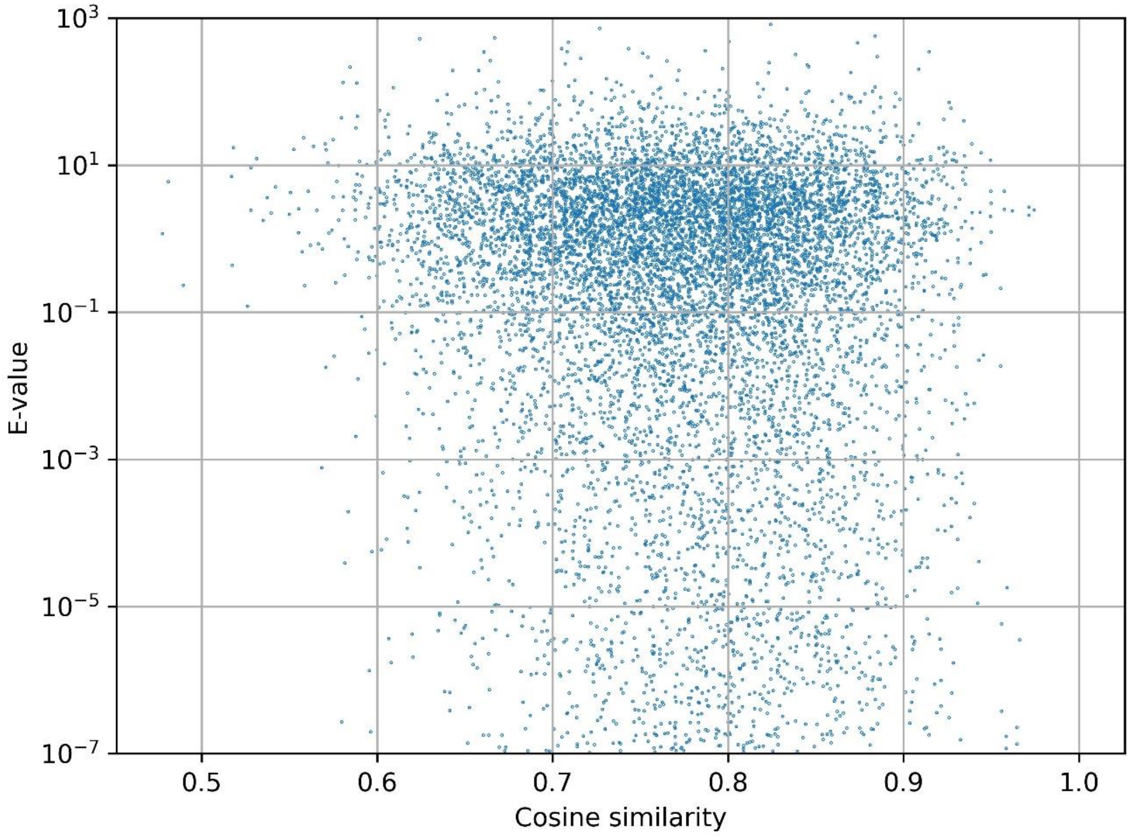

Fig. S4: CATH20 E-Value vs. Cosine Similarity. For each CATH20 query, we compare E-Value (MMseqs2) and cosine similarity (knnProtT5), showing the low correlation between the two scores

Fig. S5: Pfam20 accuracy by cosine similarity cutoff

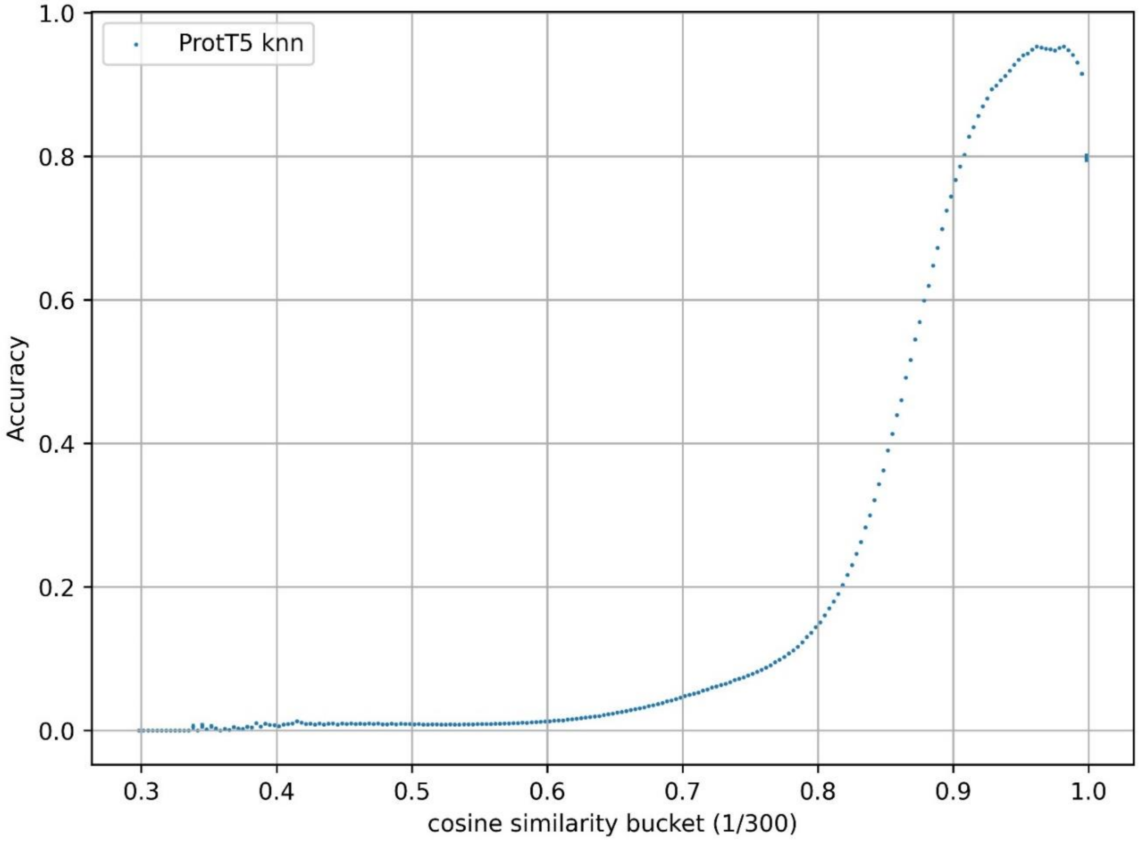

Fig. S5: Pfam20 accuracy by score cutoff. Accuracy of the knnProtT5 hits by their cosine similarity in the Pfam20 benchmark, displayed in bins of 0.01. The counterintuitive decline near 1 is due to missing annotations in Pfam: Of the 5233 matches with cosine similarity >0.95 marked as incorrect, 4577 were also found by MMseqs2 at E<0.0001, indicating they are most likely actually homologs not tracked in Pfam-A. We cannot avoid those unannotated yet correct matches since the common solution of shuffling/reversing unannotated regions is incompatible with language models (Fig. S7).

Fig. S6: Influence of number of hits for Pfam20

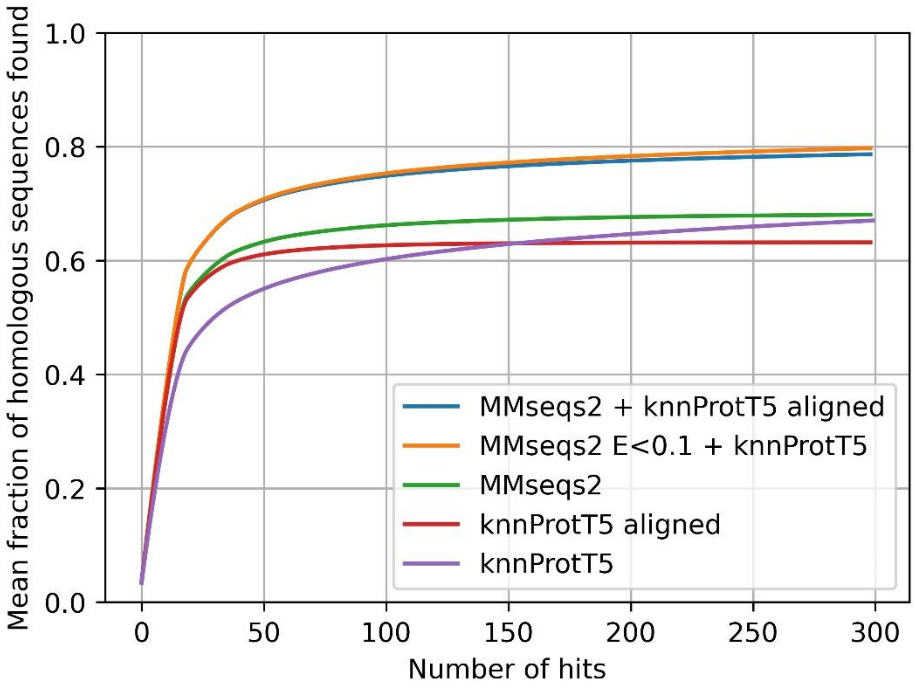

Fig. S6: Influence of number of hits for Pfam20 The fraction of homologs recovered in the Pfam benchmark when considering a certain number of hits. ProtT5 k-nn alone is bad at sorting good hits to the front, even though it recovers about the same fraction of homologs as MMseqs2 after 300 hits. After alignment, most homologs are in the first few hits, however we use some homologs in the alignment process. Simultaneously, the number of hits is the most important hyperparameter in k-nn and for a following alignment; Picking e.g. only 50 hits from ProtT5 k-nn before alignment/combining would considerably impair performance. It also shows how the combined methods outperform the others consistently.

Fig. S7: Comparing shuffled and reversed sequences

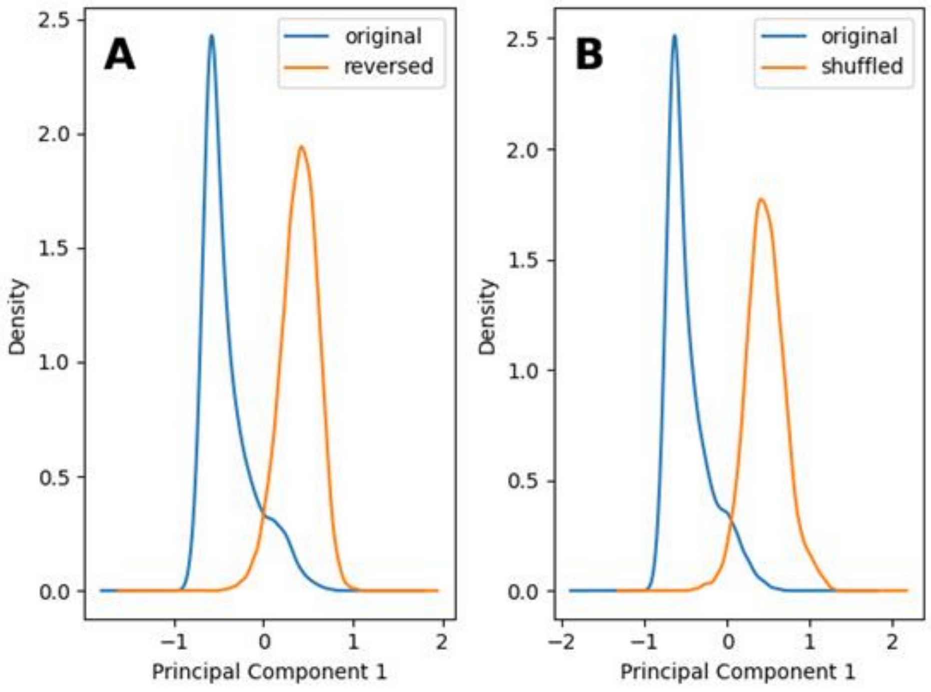

Fig. S7: Comparing shuffled and reversed sequences. We apply PCA together to embeddings of original and the same sequences together. We show Kernel Density Estimate of the first Principal Component of the PCA. **A**: original and reversed sequences. **B**: original and shuffled sequences.

Table S1: Euclidean vs. cosine CATH20 accuracies

	QnormTop1 euclidean	*QnormTop1 cosine*	*QrawTop1 euclidean*	*QrawTop1 cosine*
ProtT5 XL U50 fp16	55.3%	57.5%	70.1%	70.9%
ProtT5 XL U50	55.3%	57.5%	70.1%	70.9%
ProtT5 XL U50 L2	54.6%	56.6%	69.3%	70.3%
ProtT5 BFD	52.5%	54.3%	69.8%	70.8%
ESM1b	47.7%	47.9%	68.3%	68.5%
ESM	37.9%	43.5%	61.6%	65.2%
ProtAlbert BFD	20.0%	20.2%	34.6%	34.7%
SeqVec LSTM1	16.7%	18.6%	35.1%	37.4%
SeqVec Sum	16.7%	18.2%	35.5%	37.5%
PLUS	17.5%	17.7%	35.7%	36.0%
SeqVec LSTM2	16.5%	17.6%	35.2%	36.7%
ProtXLNet UniRef100	14.1%	15.4%	32.3%	34.2%
ProtBert BFD	11.9%	12.7%	20.2%	21.0%
UniRep	9.1%	9.1%	22.3%	22.4%
SeqVec CharCNN	2.6%	2.7%	4.2%	4.2%
AA Composition	2.5%	2.5%	3.9%	4.0%
CPCProt	1.9%	2.1%	3.8%	3.9%

*   **Table S1: Euclidean vs. cosine CATH20 accuracies.** Comparing accuracies of different language models for the CATH dataset as explained in Table 1, but also showing the euclidean distance.

Table S2: Separating fake sequences by PC1

	PC1<0	*PC1>0*
original	8856	1144
reversed	363	9637
	PC1<0	*PC1>0*
original	9162	838
shuffled	219	9781

* **Table 2: Separating fake sequences by PC1.** We apply PCA to the ProtT5 {Elnaggar, 2021 #66} embeddings of original and the same sequences scrambled together, then count the number of transformed embeddings that are smaller or larger than 0 on Principal Component 1. We do this twice, once original and reversed together and then original and shuffled together, showing the same clear separation for both.

References for Supporting Online Material

BUCHFINK, B., REUTER, K. & DROST, H.-G. 2021. Sensitive protein alignments at tree-of-life scale using DIAMOND. *Nature methods,* 18**,** 366–368.

EL-GEBALI, S., MISTRY, J., BATEMAN, A., EDDY, S. R., LUCIANI, A., POTTER, S. C., QURESHI, M., RICHARDSON, L. J., SALAZAR, G. A., SMART, A. & OTHERS 2019. The Pfam protein families database in 2019. *Nucleic acids research,* 47**,** D427–D432.

ELNAGGAR, A., HEINZINGER, M., DALLAGO, C., REHAWI, G., YU, W., JONES, L., GIBBS, T., FEHER, T., ANGERER, C., STEINEGGER, M., BHOWMIK, D. & ROST, B. 2021. ProtTrans: Towards Cracking the Language of Lifes Code Through Self-Supervised Deep Learning and High Performance Computing. *IEEE Trans Pattern Anal Mach Intell,* PP.

ORENGO, C. A., MICHIE, A. D., JONES, S., JONES, D. T., SWINDELLS, M. B. & THORNTON, J. M. 1997. CATH - a hierarchic classification of protein domain structures. *Structures,* 5**,** 1093-1108.

PETERS, M. E., NEUMANN, M., IYYER, M., GARDNER, M., CLARK, C., LEE, K. & ZETTLEMOYER, L. 2018. Deep contextualized word representations. *arXiv preprint arXiv:1802.05365*.

STEINEGGER, M. & SÖDING, J. 2017. MMseqs2 enables sensitive protein sequence searching for the analysis of massive data sets. *Nature biotechnology,* 35**,** 1026–1028.
